# Pre-pregnancy obesity and non-adherence to multivitamin use: findings from the National Pregnancy Risk Assessment Monitoring System (2009–2011)

**DOI:** 10.1186/s12884-016-1002-0

**Published:** 2016-08-05

**Authors:** Saba W. Masho, Amani Bassyouni, Susan Cha

**Affiliations:** 1 Division of Epidemiology, Department of Family Medicine and Population Health, School of Medicine, Virginia Commonwealth University, 830 E. Main Street, 8th Floor, P.O. Box 980212, Richmond, VA 23298-0212 USA; 2 Department of Obstetrics and Gynecology, School of Medicine, Virginia Commonwealth University, Richmond, VA USA; 3 Virginia Commonwealth University Institute for Women’s Health, Richmond, VA USA

**Keywords:** Obesity, Body mass index, Vitamins, Dietary supplements, Preconception care, PRAMS

## Abstract

**Background:**

Although adequate folic acid or multivitamins can prevent up to 70 % of neural tube defects, the majority of U.S. non-pregnant women of childbearing age do not use multivitamins every day. Factors influencing consistent multivitamin use are not fully explored. This study aims to investigate the association between pre-pregnancy body mass index (BMI) and multivitamin use before pregnancy using a large, nationally representative sample of women with recent live births.

**Methods:**

The national 2009–2011 Pregnancy Risk Assessment Monitoring System data were analyzed. The sample included women with recent singleton live births (*N* = 104,211). The outcome of interest was multivitamin use which was categorized as no multivitamin use, 1–3 times/week, 4–6 times/week, and daily use. Maternal BMI was examined as underweight (<18.50 kg/m^2^), normal weight (18.50–24.99 kg/m^2^), overweight (25.00–29.99 kg/m^2^), and obese (≥30.00 kg/m^2^). Multinomial logistic regression was conducted, and adjusted odds ratios and 95 % confidence intervals were calculated.

**Results:**

Compared to women with normal weight, overweight and obese women had significantly increased odds of not taking multivitamins after adjusting for confounding factors. Further, the lack of multivitamin use increased in magnitude with the level of BMI (OR_overweight_ = 1.2, 95 % CI = 1.1–1.3; OR_obese_ = 1.4, 95 % CI = 1.2–1.5).

**Conclusions:**

Obese and overweight women were less likely to follow the recommendation for preconception multivitamin use compared to normal weight women. All health care professionals must enhance preconception care with particular attention to overweight and obese women. Preconception counseling may be an opportunity to discuss healthy eating and benefits of daily multivitamin intake before pregnancy.

## Background

Multivitamin use has increased over the past decade and is the most commonly used dietary supplement in the United States [1]. According to the Centers for Disease Control and Prevention (CDC), approximately 34 % of women aged 20–39 used a dietary supplement containing folic acid and 30 % reported using supplemental vitamin D in 2003–2006 [2]. Additionally, nationally representative data that come from the Behavioral Risk Factor Surveillance System (BRFSS) indicate 78 % of pregnant women and 47 % of non-pregnant women of childbearing age (18–44) use multivitamins daily [3]. Since 1991, the U.S. Public Health Service has recommended that all women who plan on becoming pregnant consume 400 μg of folic acid daily beginning one month before trying to get pregnant [4, 5]. However, a recent report from the CDC finds that more than 63 % of women do not take multivitamins during the month before pregnancy [6]. The lack of multivitamin use is a public health concern given that adequate folic acid or multivitamins can prevent 50to 70 % of pregnancies affected by serious birth defects, (i.e., spina bifida and anencephaly) [7]. For instance, one hospital-based case–control study reported the use of folic acid (0.4 mg daily for three months before and after conception, and continuing for at least one month) was associated with decreased neural tube defects (OR = 0.32, 95 % CI = 0.17–0.58), but the protective effects of folic acid supplementation was attenuated for overweight or obese women [8]. Similarly, a recent Cochrane review, women receiving folic acid with micronutrients were less likely to have infants with neural tube defects than women receiving other or same micronutrients without folic acid (RR = 0.31, 95 % CI = 0.16–0.60; RR = 0.29, 95 % CI = 0.12–0.70) [9].

Additionally, prenatal iron deficiency anemia during pregnancy has detrimental effects on infant mental development. A cohort study that followed women during the last trimester to 24 months after delivery reported a lower mental development index among children exposed to prenatal iron deficiency anemia at 12, 18, and 24 months of age; however, supplementation with sufficient iron was found to be protective [10]. Prenatal vitamin intake during pregnancy has been shown to significantly reduce the risk of birth defects, low birth weight, small for gestational age, and spontaneous preterm birth [11–14].

Despite the benefits of multivitamins or folic acid use before pregnancy, non-adherence to recommendations has been observed in some populations. A large proportion of women who are younger (under 20 years of age), of racial/ethnic minority group, or without history of neural tube defects do not take folic acid supplements before pregnancy [15]. Other risk factors for inconsistent or no use of multivitamin during pregnancy include low income and below high school educational attainment [3, 16]. One study conducted among African-American and Hispanic women identified adverse side effects, large size of pills, and bad taste and smell as possible barriers to multivitamin use [17]. Lack of knowledge regarding multivitamin use, dose, duration, timing and efficacy can also lead to non-adherence to multivitamin use during pregnancy [18]. On the other hand, Kimmons et al. reported that obese women were less likely to use multivitamins than normal weight women [19].

To the authors’ knowledge, only a few studies have assessed the relationship between obesity and multivitamin use [20–22]. For example, a nested retrospective cohort study conducted by Farah et al. found that obese and overweight women reported less folic acid use before pregnancy compared to women of normal weight [20]. In yet another study, Case et al. used data from the Texas Behavioral Risk Factor Surveillance System (1999–2003) and found that obese but not overweight women were less likely to take folic acid every day compared to normal weight/underweight women [21]. Kimmons et al. examined the association between body mass index (BMI) and serum micronutrient levels among U.S. adults and found that premenopausal women who were overweight and obese were more likely to have low levels of micronutrients (e.g., folate) than normal weight premenopausal women [22]. Decreased folic acid intake may be due to unplanned pregnancies and failed contraceptive methods prevalent in obese women [23]. Although findings in these studies provide critical evidence, these studies have notable limitations. The study by Farah et al. had a small sample size (*n* = 288) and included only Caucasian women, making it less generalizable to minorities who may be at risk for poor pregnancy outcomes [20]. Similarly, Case et al. only considered women from Texas and excluded those who were younger than 18 years old [21]. In the study by Kimmons et al., micronutrient intake was not taken into consideration, and any adult women (≥19 years) were categorized as premenopausal if they reported menstruation in the previous 12 months, possibly including older women who were no longer of reproductive age (15–44) [22]. The observed shortcomings in the study designs, problems related to small sample sizes and homogenous study populations, warrant further investigation of the relationship between obesity and multivitamin use.

This study aims to investigate the association between pre-pregnancy BMI and multivitamins use before pregnancy using a large, nationally representative sample of women with recent live births.

## Methods

The 2009–2011 Pregnancy Risk Assessment Monitoring System (PRAMS) [24] was analyzed to assess the relationship between pre-pregnancy BMI and multivitamin use before pregnancy. PRAMS is an ongoing national surveillance program through the CDC in collaboration with participating state health departments. Data on women with recently delivered live-born infants are drawn from state birth certificate files. PRAMS utilizes a complex multistage sampling design and oversamples women from minority groups and high risk populations to ensure adequate numbers for analyses (e.g., low weight births). Accordingly, oversampling is accounted for by analysis weights provided by CDC to obtain nationally representative estimates. Women are contacted by mail or telephone to participate, and information regarding their attitudes, behaviors, and experiences before and during pregnancy, and a few months after birth are obtained from those who consent to participate. In order to ensure comparability of data, PRAMS establishes standardized surveillance methods and tools for data collection.

A total of 112,358 women (unweighted N) participated in PRAMS for 2009 to 2011. For this analysis women with singleton live births and valid responses to questions on pre-pregnancy BMI and multivitamin use a month prior to pregnancy were included. The study sample consisted of 104,211 women (unweighted number) representing 5,032,562 women (weighted number). Women who delivered multiple births (e.g., twins, triplets) were excluded from the analysis, as consistent with the literature [25, 26]. This study analyzed a de-identified publicly available data and was approved as exempt by the Institutional Review Board at Virginia Commonwealth University.

The exposure, maternal pre-pregnancy BMI was determined by self-report weight and height prior to pregnancy (kilograms/meters^2^). Participants were asked, “Just before you got pregnant with your new baby, how much did you weigh?” and “How tall are you without shoes?” Using BMI classifications from the World Health Organization, pre-pregnancy BMI was categorized as underweight (<18.5 kg/m^2^), normal weight (18.5–24.9 kg/m^2^), overweight (25.0–29.9 (kg/m^2^) and obese (≥30.0 kg/m^2^) [27].

The outcome of interest was the frequency of multivitamin use one month prior to pregnancy. Women were asked, “During the month before you got pregnant with your new baby, how many times a week did you take a multivitamin, a prenatal vitamin, or a folic acid vitamin?” Consistent with the literature, responses were categorized into four levels: “I didn’t take a multivitamins, prenatal vitamins or folic acid vitamins at all” (i.e., Not at all), “1 to 3 times a week”, “4 to 6 times a week”, or “every day of the week” [28].

Covariates examined included maternal socio-demographic factors, access to resources and care, behavioral and lifestyle factors, reproductive health factors and medical complications [15, 29–31]. Socio-demographic factors included maternal age (≤19; 20–24; 25–29; 30–35; >35), race/ethnicity (White non-Hispanic; Black non-Hispanic; Hispanic; other non-Hispanic), education (<high school; high school; >high school), marital status (married; not married), and household income level (<20,000; 20,000–34,999; 35,000–49,999; ≥50,000). Factors related to access to care and services included health insurance 12 months before pregnancy (private; Medicaid; multiple; other; none). Maternal health behaviors prior to pregnancy consisted of smoking (yes; no), drinking alcohol (yes; no), dieting, i.e., changing eating habits any time during the 12 months prior to pregnancy (yes; no), and exercise habits, i.e., exercising three or more days of the week any time during the last 12 months before pregnancy (yes; no). Maternal health conditions included preconception morbidities such as diabetes (yes; no) and hypertension (yes; no). Previous live pregnancy, pregnancy intention and intimate partner violence were also evaluated as confounders.

All analyses were conducted using SAS statistical software version 9.4 to account for the complex survey design. Descriptive statistics including unweighted frequencies and weighted percentages were generated to assess the distribution of maternal characteristics and multivitamin use. Multinomial logistic regression models for the outcome of interest were used to obtain crude and adjusted odds ratios (COR and AOR, respectively) and 95 % confidence intervals (CI). Adjusted estimates controlled for domains of covariates such as maternal socio-demographic characteristics, medical-reproductive factors, and socio-behavioral characteristics.

## Results

The majority of the study population was between the ages of 20–29 years (53 %), Non-Hispanic White (60 %), married (61 %), reported high school or more education (58 %), earned less than $50,000 (64 %), and privately insured (56 %) (Table 1). Approximately 4.4 % of women were underweight, 50.4 % were normal weight, 24.1 % were overweight, and 21.2 % obese. More than half of women (55.4 %) did not take multivitamins at any time, 8.4 % used multivitamins 1–3 times per week, 6.1 % used multivitamins 4–6 times per week, and 30.2 % used multivitamins every day. Multivitamin intake was strongly associated with socio-demographic characteristics (e.g., marital status, income, race/ethnicity), medical-reproductive (e.g., parity, pre-pregnancy diabetes), and social-behavioral factors (e.g., smoking prior to pregnancy, intimate partner violence) (Table 2). Pre-pregnancy BMI was significantly associated with age, race, marital status, education, income, insurance, parity, pre-pregnancy diabetes, pre-pregnancy hypertension, pregnancy intention, smoking prior to pregnancy, alcohol use, exercise, dieting, and intimate partner violence (Table 3).

**Table 1:** Distribution of the study population characteristics

	Total Weighted *N* = 5 032 562
	%
Age	
≤ 19	9.3
20–24	22.9
25–29	29.7
30–35	24.7
> 35	13.9
Race/Ethnicity	
White, non-Hispanic	60.4
Black, non-Hispanic	13.7
Hispanic	17.8
Other, non-Hispanic	8.2
Married	61.0
Education	
< High school	15.8
High school	26.3
> High school	57.9
Income	
< $20,000	36.2
$20,000–34,999	17.0
$35,000–49,999	10.3
v ≥ $50,000	36.5
Pre-pregnancy Insurance	
Private	55.9
Medicaid	15.6
Other	1.9
Multiple	3.4
No insurance	23.2
Previous live birth	
0	42.0
v1 - 2	47.5
≥ 3	10.6
Pre-pregnancy diabetes	2.2
Pre-pregnancy hypertension	8.9
Unintended pregnancy	42.5
Pre-pregnancy smoking	24.6
Average number of alcohol drink/week	
None	17.0
Up to 3	67.7
4 or more	15.3
Pre-pregnancy dieting	28.5
Pre-pregnancy exercising	42.2
Intimate partner violence	3.0

Weighted *N* = adjusted for the complex sampling design and represents the total population that the sampled was derived from

**Table 2:** Prevalence of multivitamin intake by maternal characteristics

	No intake *n* = 2 946 523	1–3 times/week *n* = 444 399	4–6 times/week *n* = 323 867	Every day *n* = 1 607 653	*P*-value
Weighted row %					
Body mass index					<.0001
Underweight	61.6	8.5	4.6	25.3	
Normal Weight	51.09	8.50	6.92	33.49	
Overweight	56.47	8.33	6.07	29.13	
Obese	60.9	8.3	5.0	25.8	
Socio-demographic					
Age					<.0001
≤ 19	77.6	5.6	1.9	14.9	
20–24	71.8	7.3	3.8	17.1	
25─29	54.3	8.7	6.2	30.8	
30–35	42.3	9.2	8.0	40.5	
> 35	38.5	9.8	8.9	42.8	
Race/Ethnicity					<.0001
White, non-Hispanic	49.2	8.6	7.5	34.7	
Black, non-Hispanic	66.6	9.1	3.5	20.8	
Hispanic	66.0	7.2	3.9	22.9	
Other, non-Hispanic	54.1	8.2	6.1	31.6	
Married					<.0001
Yes	43.5	9.2	8.2	39.1	
Other	73.2	7.1	2.9	16.8	
Education					<.0001
< High school	71.0	6.7	2.9	19.4	
High school	68.6	7.1	3.6	20.7	
> High school	44.2	9.5	8.2	38.1	
Income					<.0001
< $20,000	72.2	7.5	3.1	17.2	
$20,000–34,999	62.7	8.6	5.2	23.5	
$35,000–49,999	53.1	9.6	7.6	29.7	
≥ $50,000	33.0	9.0	9.8	48.2	
Pre-pregnancy Insurance					<.0001
Private	42.8	9.0	8.2	40.0	
Medicaid	66.8	8.1	3.6	21.5	
Other	61.3	8.7	4.5	25.5	
Multiple	57.4	8.1	3.9	30.6	
No insurance	73.5	7.2	3.6	15.7	
Medical-reproductive					
Previous live birth					<.0001
0	54.8	7.1	5.4	32.7	
1–2	54.6	9.0	6.6	29.8	
≥ 3	60.94	10.1	6.5	22.54	
Pre-pregnancy diabetes					<.0001
No	55.5	8.3	6.1	30.1	
Yes	50.9	8.6	4.7	35.8	
Pre-pregnancy hypertension					<.0001
No	56.1	8.3	6.2	29.4	
Yes	47.3	8.7	4.9	39.1	
Pregnancy intention					<.0001
Unintended	71.9	8.1	4.00	16.0	
Intended	43.0	8.5	7.6	40.9	
Socio-behavioral					
Pre-pregnancy smoking					<.0001
No	49.9	8.8	6.8	34.5	
Yes	72.5	6.9	3.9	16.7	
Average # of alcohol drink/week					<.0001
None	55.1	6.9	5.2	32.8	
Up to 3	52.5	8.6	7.2	31.7	
v4 or more	58.5	8.1	7.0	26.4	
Pre-pregnancy dieting					<.0001
No	57.2	7.8	5.7	29.3	
Yes	50.6	9.8	7.1	32.5	
Pre-pregnancy exercising					<.0001
No	63.1	7.8	4.8	24.3	
Yes	44.5	9.0	7.9	38.6	
Intimate partner violence					<.0001
No	54.6	8.4	6.2	30.8	
Yes	72.9	7.4	3.1	16.6	

Weighted *N* = adjusted for the complex sampling design and represents the total population that the sampled was derived from

**Table 3:** Maternal characteristics by Body Mass Index

	Underweight *n* = 219 560	Normal Weight *n* = 2 534 187	Overweight *n* = 1 212 554	Obese *n* = 1 066 261	*P*-value
Weighted row %					
Socio-demographic					
Age					<.0001
≤ 19	17.4	10.9	7.4	5.8	
20–24	30.9	22.2	23.1	22.7	
25–29	26.7	28.5	29.8	31.0	
30–35	16.8	25.0	24.7	25.4	
> 35	8.3	13.4	15.0	15.1	
Race/Ethnicity					<.0001
White, non-Hispanic	60.4	63.7	56.0	56.2	
Black, non-Hispanic	11.7	10.8	15.6	18.7	
Hispanic	14.8	16.0	20.4	19.8	
Other, non-Hispanic	13.1	9.4	7.1	5.4	
Married					<.0001
Yes	52.2	63.5	60.5	57.4	
Other	47.8	36.5	39.5	42.6	
Education					<.0001
< High school	22.2	15.1	16.1	16.0	
High school	29.4	23.6	27.2	31.2	
> High school	48.4	61.4	56.6	52.9	
Income					<.0001
< $20,000	48.9	32.9	36.7	40.8	
$20,000–34,999	15.0	15.4	17.7	20.4	
$35,000–49,999	9.1	9.8	10.6	11.3	
≥ $50,000	27.0	41.9	35.1	27.5	
Pre-pregnancy Insurance					<.0001
Private	45.3	60.0	54.8	49.3	
Medicaid	20.9	13.9	15.2	19.1	
Other	2.11	1.9	1.9	2.1	
Multiple	3.6	3.2	3.3	3.9	
No insurance	28.0	21.0	24.8	25.6	
Medical-reproductive					
Previous live birth					<.0001
0	51.6	45.8	38.7	34.7	
1–2	40.8	45.7	49.0	51.4	
≥ 3	7.6	8.6	12.3	13.9	
Pre-pregnancy diabetes	1.6	1.4	2.2	3.9	<.0001
Pre-pregnancy hypertension	7.1	7.1	9.0	13.3	<.0001
Unintended Pregnancy	50.0	40.6	42.0	45.9	<.0001
Socio-behavioral					
Pre-pregnancy smoking	30.7	22.5	24.5	28.3	<.0001
Average # of alcohol drink/week					<.0001
None	17.7	15.6	17.2	19.8	
Up to 3	68.9	67.5	67.3	68.3	
4 or more	11.4	15.0	13.4	10.3	
Pre-pregnancy dieting	8.1	21.0	36.5	41.4	<.0001
Pre-pregnancy exercising	31.0	45.8	42.8	35.3	<.0001
Intimate partner violence	4.4	2.8	3.2	3.2	0.0085

Weighted *N* = adjusted for the complex sampling design and represents the total population that the sampled was derived from

The unadjusted analyses showed a statistically significant association between pre-pregnancy BMI and multivitamin use (Table 4). Underweight and obese women were 1.6 times as likely to report no multivitamin use compared to women of normal weight (COR = 1.6, 95 % CI = 1.4–1.8; COR = 1.6, 95 % CI = 1.5–1.7, respectively). In addition, overweight women had 1.3 times the odds of reporting no intake compared to women with normal BMI (COR = 1.3, 95 % CI = 1.2–1.4). When controlling for socio-demographic characteristics such as maternal age, race, marital status, education, income, and health insurance, the odds of non-adherence to multivitamin use stayed significant for overweight and obese women (AOR = 1.2, 95 % CI = 1.1–1.3; AOR = 1.4, 95 % CI = 1.3–1.5, respectively). However, estimates were no longer significant for the underweight group (AOR = 1.2, 95 % CI = 1.0–1.4). After additionally adjusting for medical-reproductive factors such as previous live birth, pregnancy intention, pre-pregnancy diabetes, and pre-pregnancy hypertension, the estimates remained consistent for overweight (AOR = 1.2, 95 % CI = 1.2–1.3) and obese women (AOR = 1.5, 95 % CI = 1.03–1.6). In fact, overweight and obese women were significantly more likely to report no multivitamin use compared to normal weight women (AOR = 1.2, 95 % CI = 1.1–1.3; AOR = 1.4, 95 % CI = 1.2–1.5, respectively) even after controlling for all covariates including socio-demographic characteristics, medical-reproductive factors, and socio-behavioral characteristics (i.e., smoking, alcohol consumption, diet, exercise, and intimate partner violence).

**Table 4:** Adjusted logistic regression models for non-adherence to multivitamin use among BMI Groups

	Underweight	Overweight	Obese
COR^a^			
No intake	**1.6 (1.4–1.8)**	**1.3 (1.2–1.4)**	**1.6 (1.5–1.7)**
1–3 times/week	1.3 (1.0–1.6)	1.1 (1.0–1.2)	**1.3 (1.1–1.4)**
4–6 times/week	0.9 (0.7–1.1)	1.0 (0.9–1.1)	0.9 (0.8–1.1)
AOR^b^			
No intake	1.2 (1.0–1.4)	**1.2 (1.1–1.3)**	**1.4 (1.3–1.5)**
1–3 times/week	1.2 (1.0–1.5)	1.1 (1.0–1.2)	1.2 (1.0–1.3)
4–6 times/week	1.0 (0.8–1.2)	1.0 (0.9–1.1)	1.0 (0.9–1.1)
AOR^c^			
No intake	1.2 (1.0–1.4)	**1.2 (1.2–1.3)**	**1.5 (1.3–1.6)**
1–3 times/week	1.2 (1.0–1.5)	1.1 (1.0–1.2)	**1.2 (1.1–1.3)**
4–6 times/week	1.0 (0.8–1.3)	1.0 (0.9–1.1)	1.0 (0.9–1.2)
AOR^d^			
No intake	1.1 (0.9–1.4)	**1.2 (1.1–1.3)**	**1.4 (1.2–1.5)**
1–3 times/week	1.4 (1.0–1.8)	1.1 (0.9–1.2)	1.1 (0.9–1.3)
4–6 times/week	1.1 (0.8–1.5)	1.0 (0.9–1.2)	1.0 (0.9–1.2)

Daily intake is referent outcome group; bolded estimates indicate statistical significance

^a^Crude odds ratio of multivitamin intake

^b^Adjusted for socio-demographic characteristics (age, marital status, race/ethnicity, education, income, and health insurance)

^c^Adjusted for socio-demographic characteristics and medical-reproductive factors (previous live birth, pregnancy intention, pre-pregnancy diabetes, and pre-pregnancy hypertension)

^d^Adjusted for socio-demographic characteristics, medical-reproductive factors, and socio-behavioral characteristics (pre-pregnancy smoking, average # of alcohol drink per week, pre-pregnancy dieting, pre-pregnancy exercising, and intimate partner violence)

The odds of inadequate multivitamin supplementation, specifically 1–3 times per week, were higher for obese women compared to normal weight women (COR = 1.3, 95 % CI = 1.1–1.4). Similar estimates were obtained after adjusting for socio-demographic and medical-reproductive factors (AOR = 1.2, 95 % CI = 1.1–1.3). However, there were no longer significant differences between obese and normal weight women in fully adjusted models (AOR = 1.1, 95 % CI = 0.9–1.3). The odds of multivitamin use (4–6 times per week) remained non-significant for all different weight groups, even after controlling for all aforementioned factors.

## Discussion

We found that obese and overweight women were less likely to follow the recommended use of preconception multivitamins compared to normal weight women. These findings are consistent with previous studies [20, 21]. Unlike previous studies, this analysis employed data from a national survey that provided a large sample size and representative study population. For instance, contrary to findings from Farah et al. which were based on a small sample of Caucasian women, the current study provided estimates that are more generalizable to the U.S. population [20].

Adequate multivitamin use one month prior to pregnancy has well-known positive benefits for both mothers and developing infants. However, the CDC reported that 63 % of women do not take the recommended amount of multivitamin one month prior to pregnancy [6]. This is consistent with our study finding that more than half of the women did not take multivitamin during the preconception period. While there are several possible reasons for lack of multivitamin use (e.g., simply forgetting), a growing body of literature has attributed this lack of adherence to unintended pregnancies [32–34]. Previous studies reported that obese women have higher rates of unintended pregnancies compared to their normal weight counterpart. Huber et al. analyzed PRAMS database and reported that obese women are at higher risk of unintended pregnancy compared to normal weight women (AOR = 1.75, 95 % CI = 1.21–2.52) [33]. Similarly, Garbers et al. found that obese women had more than twice the odds of unintended pregnancy (AOR = 2.81, 95 % CI = 1.41–5.60) [35]. Nevertheless, findings from the current study demonstrated that obese women had a higher odds of not using multivitamins even after adjusting for pregnancy intention.

Another possible explanation for the association between obesity and lack of multivitamin use may be that overweight and obese women are less likely to take dietary supplements. This was demonstrated by Radimer et al. who reported that obese adults were less likely to engage in healthy behaviors including multivitamin use [36]. Furthermore, it is also possible that overweight and obese women may not receive the appropriate counseling for preconception multivitamin use. The CDC reports that obese/overweight women are more likely to receive suboptimal preconception care in contrast to normal weight women [6]. Health care providers may play an important role in multivitamin use prior to pregnancy. However, only one of six obstetrician/gynecologists or family physicians provides preconception care to prenatal care patients [37].

The strengths of this study consist of having a large nationally-representative data that utilizes standardized methods of data collection. These methods allow for optimal comparison among participating states. The PRAMS data also allowed this study to account for important socio-demographic, medical, and high-risk behavioral factors. Despite these strengths, the study has some limitations. The cross-sectional design makes it difficult to determine causality between exposure and outcome. However, this study included women who had a live birth and the question about preconception multivitamin use clearly referred to one month prior to pregnancy. Another limitation of the study is that BMI and multivitamin use were both self-reported. It is possible that overweight and obese women may be under-reporting their weight because of social desirability bias which may bias the estimate toward the null. Additionally, PRAMS questionnaire is administered several months after delivery, making women’s answers subject to recall bias. However, limiting the recall period to no longer than one year might minimize recall bias. In fact, PRAMS typically contacts women two to four months after delivery [38, 39]. Lastly, the effect of preconception counseling on multivitamin use may have affected the results but were unavailable in the data. Health education and counseling on positive health behaviors during perinatal care visits may be of interest for future research.

## Conclusions

In summary, overweight and obese women are less likely to follow the universal recommendation for multivitamin use one month before pregnancy. This behavior might increase the risk for serious but preventable outcomes in infants, such as neural tube defects, anencephaly, multiple birth defects, small for gestation, and low birth weight. More research and well-designed studies are needed to investigate in detail the reasons for inadequate multivitamin use in obese/overweight women during the preconception period. Knowing the motivations underlying this problem would help health policy makers implement a proper intervention. However, health care providers and policy makers need to raise awareness of this significant health problem. Furthermore, all health care professional should enhance preconception care during well–woman visits or other routine visits, and particular attention should be given to obese women. Preconception counseling presents an opportunity to discuss healthy eating and healthy weight, as well as the benefit of daily multivitamin intake before pregnancy.

## Abbreviations

AOR, adjusted odds ratio; BMI, body mass index; BRFSS, behavioral risk factor surveillance system; CDC, centers for disease control and prevention; CI, confidence interval; COR, crude odds ratio; OR, odds ratio; PRAMS, pregnancy risk assessment monitoring system
